# Vesicles Balance Osmotic Stress with Bending Energy That Can Be Released to Form Daughter Vesicles

**DOI:** 10.1021/acs.jpclett.1c03369

**Published:** 2022-01-10

**Authors:** Xiaoyan Liu, Joakim Stenhammar, Håkan Wennerström, Emma Sparr

**Affiliations:** Physical Chemistry, Lund University, 221 00 Lund, Sweden

## Abstract

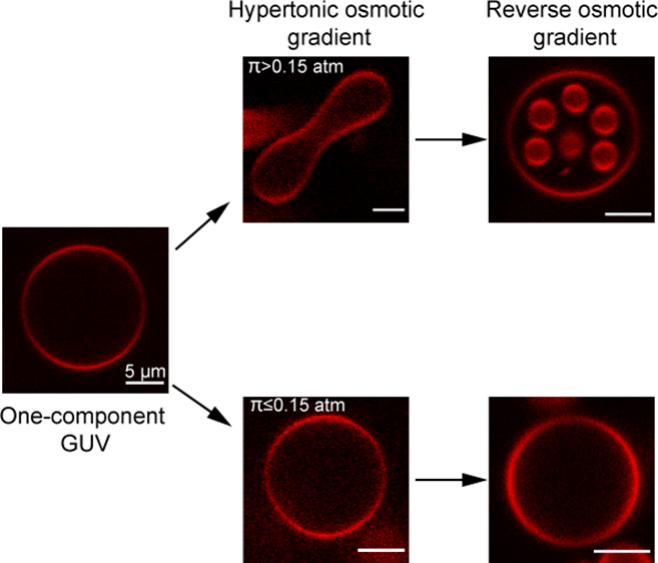

The bending energy of the lipid membrane is central to biological processes involving vesicles, such as endocytosis and exocytosis. To illustrate the role of bending energy in these processes, we study the response of single-component giant unilamellar vesicles (GUVs) subjected to external osmotic stress by glucose addition. For osmotic pressures exceeding 0.15 atm, an abrupt shape change from spherical to prolate occurs, showing that the osmotic pressure is balanced by the free energy of membrane bending. After equilibration, the external glucose solution was exchanged for pure water, yielding rapid formation of monodisperse daughter vesicles inside the GUVs through an endocytosis-like process. Our theoretical analysis shows that this process requires significant free energies stored in the deformed membrane to be kinetically allowed. The results indicate that bending energies stored in GUVs are much higher than previously implicated, with potential consequences for vesicle fusion/fission and the osmotic regulation in living cells.

Much of the material transport within and between cells involves lipid vesicles. The key processes are lipid membrane fusion and fission, typically described as exocytosis and endocytosis when occurring in a cellular environment. These processes play a central functional role in living systems and are controlled by complex mechanisms evolved through natural selection. In the cellular environment, endocytosis and exocytosis can be triggered in a number of ways depending on the local conditions.^1,2^ However, in all cases, the basic molecular event is a vesicle fusion or fission that changes the topology of the lipid membrane. For such a process to occur, it has to be kinetically allowed, i.e., having a sufficiently low energy barrier, while the direction (fission or fusion) is determined by global free energy minimization. One route toward a molecular understanding of exocytosis and endocytosis is to study the fundamental aspects of the phenomenon and to clarify obstacles that must be overcome in specific physiological processes in order to accomplish a specific event. One key open question here is the role of different membrane-binding proteins in promoting fusion or fission relevant for living systems.^3−7^ Another important discussion concerns the fusion or fission of essentially pure lipid vesicles, where no such proteins are present.^8,9^ The membrane remodeling processes associated with membrane fusion, fission, and tubulation all involve the formation of highly curved structures and may therefore be facilitated by an asymmetric or patchy organization of lipids and proteins in the membrane.^10−14^ Vesicle deformation and fission can furthermore be induced by changes in external conditions that may involve chemical gradients across the membrane^15,16^ or temperature changes.^17−19^

Another physiologically relevant aspect affecting the properties of cellular membranes is the regulation of osmotic pressure. An imbalance in osmotic pressure between the extracellular and intracellular regions will drive water transport and can furthermore promote membrane remodeling, including fission, membrane deformation, and tubulation.^15,16,20−26^ For a majority of cells, whether prokaryotic or eukaryotic, optimal growth conditions occur for osmotic pressures in the range between 7 and 10 atm (0.25 to 0.4 osmol).^27^ Living organisms have developed a number of strategies to help maintain an optimal intracellular concentration of proteins and other molecular components even when the external osmotic pressure deviates from the preferred value. Most bacteria have a double cell wall, and at low external osmotic pressures, a turgor pressure develops to prevent water from diffusing into the cell.^28^ In environments with high osmotic pressures, such as the ocean, the simplest way to increase the internal osmotic pressure is to increase the intracellular electrolyte concentration, which can however perturb the intermolecular interactions between the components of the cell. An alternative and more versatile method is therefore to instead import or synthesize electrically neutral osmolytes to obtain a balanced osmotic pressure.^29−31^

In the present paper, we study the response of single-component giant unilamellar vesicles, GUVs, to changes in osmotic conditions. Fluorescently labeled vesicles, monitored by confocal microscopy, are initially prepared in pure water and then exposed to variations in the osmotic pressure of the external medium. We follow the osmotically induced shape changes of the GUVs and show how an increase in the external osmotic pressure can induce a global deformation of pure lipid vesicles. While this observation is in qualitative accordance with theoretical predictions based on the membrane bending energy,^32,33^ the deformation occurs at osmotic pressures several orders of magnitude higher than predicted, indicating that the associated membrane bending energy is much larger than the thermal energy. Upon reversing the osmotic gradient, we furthermore demonstrate a spontaneous fission process leading to the formation of daughter vesicles inside the primary mother vesicle. These results demonstrate a clear coupling between osmotic changes and vesicle fission, and an energetic analysis of the fission process confirms that the bending energy stored in the bent lipid membrane is orders of magnitude larger than previously implied.

A number of previous experimental and computational studies have demonstrated the formation of daughter vesicles^15,16,20,22,34^ and vesicle deformation^15,20,21,24,35^ in response to osmotic stress. In several of these, it was shown that fission can be induced by hypertonic osmotic gradients, although typically at values considerably higher than those used here.^15,16,21^ In the majority of previous studies, the vesicles were composed of more than a single lipid component and often with added solutes both inside and outside.^15,16,20,22,34,36^ Several of these previous studies have focused on the *dynamics* of deformation rather than the stable (quasi-)equilibrium situation, which can take minutes or longer to reach depending on the vesicle size and the magnitude of the osmotic gradient. Our results are fully compatible with earlier observations; however, we have made an effort to reduce the complexity of the system as much as possible by studying vesicles composed of a single (or, in some cases, two) lipid components, diffusing freely in a bulk solution containing no additional solutes such as buffer or electrolytes. As we will demonstrate below, this simplicity enables theoretical analysis of the observed phenomena and allows us to shed light on the energetics of vesicle deformation and fission.

*Basic Observations*. Giant unilamellar vesicles (GUVs) were prepared by using the electroformation method^37^ using a homemade fluidic flow channel with indium tin oxide (ITO)-coated glass slides,^38^ allowing for *in situ* observations directly after changing solution conditions. Due to the narrow channels, it is possible to image free single vesicles over time. GUVs composed of a single lipid component, DOPC, were prepared in pure water with no added buffer or solutes. The GUVs were then put under osmotic stress by adding glucose to the bulk phase (Figure 1). The response of the GUV was monitored using confocal microscopy by having a small fraction (0.5 mol %) of the lipids labeled by a fluorophore (Lissamine rhodamine B, red). As shown in Figure 1b, the DOPC GUVs keep their apparent spherical shape at an external glucose concentration of 4 mM, corresponding to an osmotic pressure of 0.1 atm. However, for a 3 times higher glucose concentration, the GUVs rapidly (within 2 min) become deformed to a smooth prolate shape as shown in Figures 1e,h and S1. After this relatively rapid deformation, the shape of the GUVs remains stable for several hours if the solution conditions are unchanged. In a next set of experiments, we reversed the osmotic gradient, by rinsing with pure water (Figure 1c,f,i). The response of the GUVs to the rinsing step was dramatically different depending on the magnitude of the initial osmotic stress (0.1 or 0.3 atm). In the former case, rinsing led to no visible changes (Figure 1c), while in the latter case, the rinsing resulted in the formation of internal “daughter” vesicles having radii approximately a factor of 5 smaller than the original GUVs (Figure 1f). By adding a green water-soluble fluorescent molecule (Alexa488) to the rinsing water, it is clear that the interior of the daughter vesicles emanates from the bulk liquid (Figure 1f). We furthermore observe that the daughter vesicles appear to be fully disconnected from the parent lipid bilayer (Figure S2 as well as Movies S1 and S2). On the other hand, when the lipid membranes are made more permeable to water by the addition of the antimicrobial bee venom peptide melittin,^39−41^ no daughter vesicles form inside the GUVs, but the mother GUVs are instead filled by the green fluorescent dye (Figure 1i). In a separate experiment, we confirmed that melittin permeabilizes the lipid membranes to Alexa488 also in the absence of an osmotic gradient (Figure S3). The contribution from possible contamination solutes in the Milli-Q water (including release of ions from glassware) was estimated to be below 10 μM, in line with previous measurements.^42^ Finally, we again stress that the present results only concern freely suspended vesicles in order to avoid surface-induced vesicle deformation that may occur for anchored and sedimented vesicles.^16,22,34,43^

**Figure 1:**
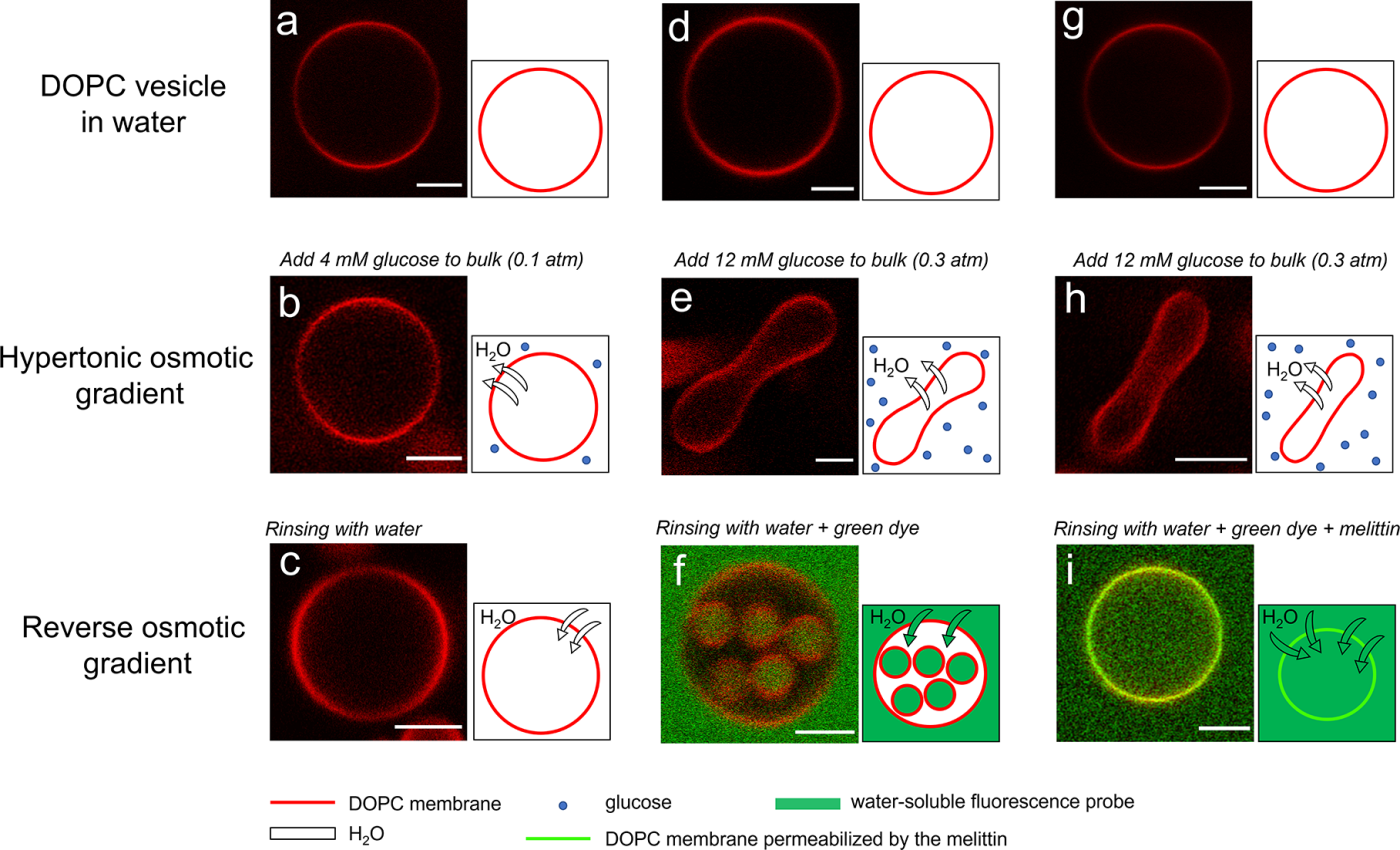
2D CLSM images of DOPC vesicles in pure water (a,d,g), exposed to osmotic gradients of 0.1 atm (b) or 0.3 atm (e,h) and subsequently rinsed by water (c,f) or water with 1 μM melittin (i). For panels f and i, a water-soluble green fluorophore Alexa488 was added to the rinsing water. The vesicles diffused freely in the microfluidic channel and were not in contact with any surface. The figure shows representative images for the different conditions and does not depict the same vesicle at different steps of the experiments. The temperature was kept constant at 20 °C, and the scale bars represent 5 μm.

A number of control experiments were performed to confirm the observations described above. First, the experiments were repeated with GUVs composed of only DOPC and no fluorescent lipid, showing the same response to the changes in the osmotic gradient as for the labeled vesicles (Figure S4). Second, the experiments were repeated with a water-soluble polymer, PEG2000, as a solute instead of glucose (Figure S5), again showing the same behavior as depicted in Figure 1 with vesicle deformation and formation of daughter vesicles in response to the variations in the osmotic gradient. These experiments exclude that the observations made in Figure 1 are due to specific interactions between the lipid headgroups and glucose. Moreover, as shown in Figure S6, Alexa488 was added to the outside solution containing deformed vesicles at an osmotic gradient of 0.3 atm, and we did not observe any dye inside the vesicles, indicating that the membrane barrier properties remain after deformation.

The observations in Figure 1 raise a number of questions that we discuss in detail below:

(1) When a vesicle or a cell is exposed to an osmotic imbalance, it is normally assumed that the induced transport of water across the bilayer results in a restoration of matching solute concentrations. The addition of glucose to the bulk induces a net diffusion of water from the GUV interior to its exterior. However, in the case shown in Figure 1b, there is no solute inside the GUV. Nevertheless, it still retains a volume and shape visually identical with that before the application of the osmotic gradient. The result presented in Figure 1e,h shows that water diffusion can occur across the bilayer, as expected from the known water permeability coefficient.^44^ Do the observed vesicle shapes represent an equilibrium with respect to water diffusion across the bilayer, and if so, what is the mechanism for establishing this equilibrium?
(2) What is the microscopic mechanism causing the observed qualitative change in response between the cases of low (0.1 atm, Figure 1b) and high (0.3 atm, Figure 1e,h) osmotic stresses?
(3) When there is a visible osmotically induced shape change of the vesicles, they retain a smooth prolate envelope, and the bilayer shape changes only on a length scale comparable to the radius of the GUV. Is this observed deformation in qualitative and quantitative accordance with previous theoretical predictions^33,45^ based on membrane bending energies?
(4) For a symmetric bilayer, the vesicle fission process leading to the formation of daughter vesicles in Figure 1f is normally a thermodynamically very unfavorable process for symmetric bilayers. What makes it thermodynamically and kinetically allowed under the given circumstances, and what makes the endocytosis process favored over exocytosis?
(5) There is a qualitative difference in response to the restoration of osmotic conditions between the systems in Figures 1c, 1f, and 1i. Does this difference have a thermodynamic or kinetic basis?

*Vesicles under Hypertonic Stress*. When glucose is added to the GUV suspension, a nonequilibrium situation is established, where the chemical potential of water is lower in the bulk solution than inside the vesicles, equivalent to a difference in osmotic pressure across the lipid bilayer. There is thus a driving force for water diffusion from the inside to the outside, with a corresponding driving force for glucose diffusion from the outside into the vesicle. However, the permeability across the bilayer is around 6 orders of magnitude larger for water than for glucose,^46,47^ so that the kinetically dominant process is the water flux. The osmotic pressure, π, is usually expressed in terms of the solute concentration, *c*_*s*_, through the van’t Hoff equation
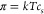
1where *k* is Boltzmann’s constant and *T* is the temperature. This equation assumes that the dominant concentration-dependent contribution to the free energy is due to ideal entropy of mixing. In a more general treatment, π is directly related to the chemical potential of water, *μ*_*w*_, through the product with the molecular volume of water, *V*_*w*_, so that
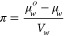
2where μ_*w*_^*ο*^ is the standard chemical potential of pure water.

When glucose is added to the bulk solution, water diffuses out of the GUVs, and the enclosed vesicle volume decreases, while the number of lipids in the membrane remains constant. The lipid bilayer is essentially incompressible in the lateral direction for the present range of osmotic pressures,^48^ and to remain at constant area, the vesicle thus has to buckle. This in turn increases the bending free energy of the bilayer. Each bilayer configuration is associated with a bending energy *E*_*b*_, which, to leading order in the curvature, is given by Helfrich’s expression^49^
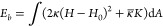
3Here, κ is the bending rigidity of the bilayer, *H* is its mean curvature, *H*_0_ is the spontaneous curvature, κ̅ is the saddle splay constant, *K* is the Gaussian curvature, and the integral runs over the area *A* of the bilayer envelope. For finite temperatures, the vesicle shape fluctuates due to thermal excitations, and to have a complete description of the system, it is necessary to average over these fluctuations.^56^ The corresponding free energy *F*_*b*_ is then given by
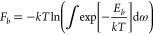
4where the integral runs over all thermally accessible configurations of the vesicle shell. The free energy contribution from the bending fluctuations is often ignored when discussing vesicle shapes^50^ but can play a central role in bilayer systems, for example resulting in long-ranged undulation forces between lipid bilayers.^51^ The chemical potential of water μ_*w*_^*i*^ inside the vesicle volume *V*_0_ containing *n*_*w*_^*i*^ = *V*_0_/*V*_*w*_ water molecules and no solute is furthermore given by
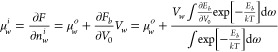
5At equilibrium with respect to water transport, the chemical potentials inside and outside the vesicle are equal, and assuming ideal mixing in the bulk gives, by combining eqs 1, 2, and 5,
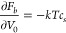
6The above reasoning shows that the vesicle bending energy contributes to the chemical potential of water and thus also to the osmotic pressure.^50^ A detailed calculation of the balance between osmotic pressure and bending energy in the athermal (*T* = 0) case however indicates that this effect gives a negligible contribution that can be ignored for practical purposes,^49^ as we discuss in more detail below.

In experiments, we observe a rapid deformation of the GUVs when glucose is added to the bulk, after which the GUV shape remains stable for an extended period. The most straightforward explanation of this observation is that the equilibrium of eq 6 has been established, where the free energy associated with the mixing entropy of water and glucose in the bulk is balanced by the bending free energy of the vesicle bilayer. To further establish that this is indeed the case, experiments were performed for GUVs at different values of the osmotic stress and for three lipid systems with distinctly different bending rigidities. Figure 2a–e shows that the deformation at (quasi-) equilibrium increases with increasing bulk osmotic pressure, qualitatively consistent with eqs 4–6. Comparing panels for bulk osmotic pressures of 0.2 atm (c,i,o), 0.3 atm (d,j,p), and 0.4 atm (e,k,q), respectively, shows consistently that, at a given bulk osmotic pressure, the vesicle deformation is largest for pure DOPC vesicles, followed by vesicles composed of DMPC with 5 mol % cholesterol, while the smallest deformation is observed for vesicles composed of DMPC with 30 mol % cholesterol. Pure DMPC bilayers with saturated C14 chains have a bending rigidity κ ≈ 29 *kT* (*T* > 24 °C),^52^ somewhat larger than the bending rigidity of κ ≈ 20 *kT* for DOPC with unsaturated chains.^53^ Cholesterol causes a straightening of the chains and an increase in bending rigidity.^52,54^ The bilayer composed of DMPC and 30 mol % cholesterol forms a liquid ordered lamellar phase^55^ with a considerably higher bending rigidity of κ ≈ 97 *kT*.^52^Figure 2 thus directly demonstrates that vesicle deformations decrease with increasing membrane rigidity, indicating that the observed deformation is indeed controlled by an energetic balance between membrane bending and osmotic stress.

**Figure 2:**
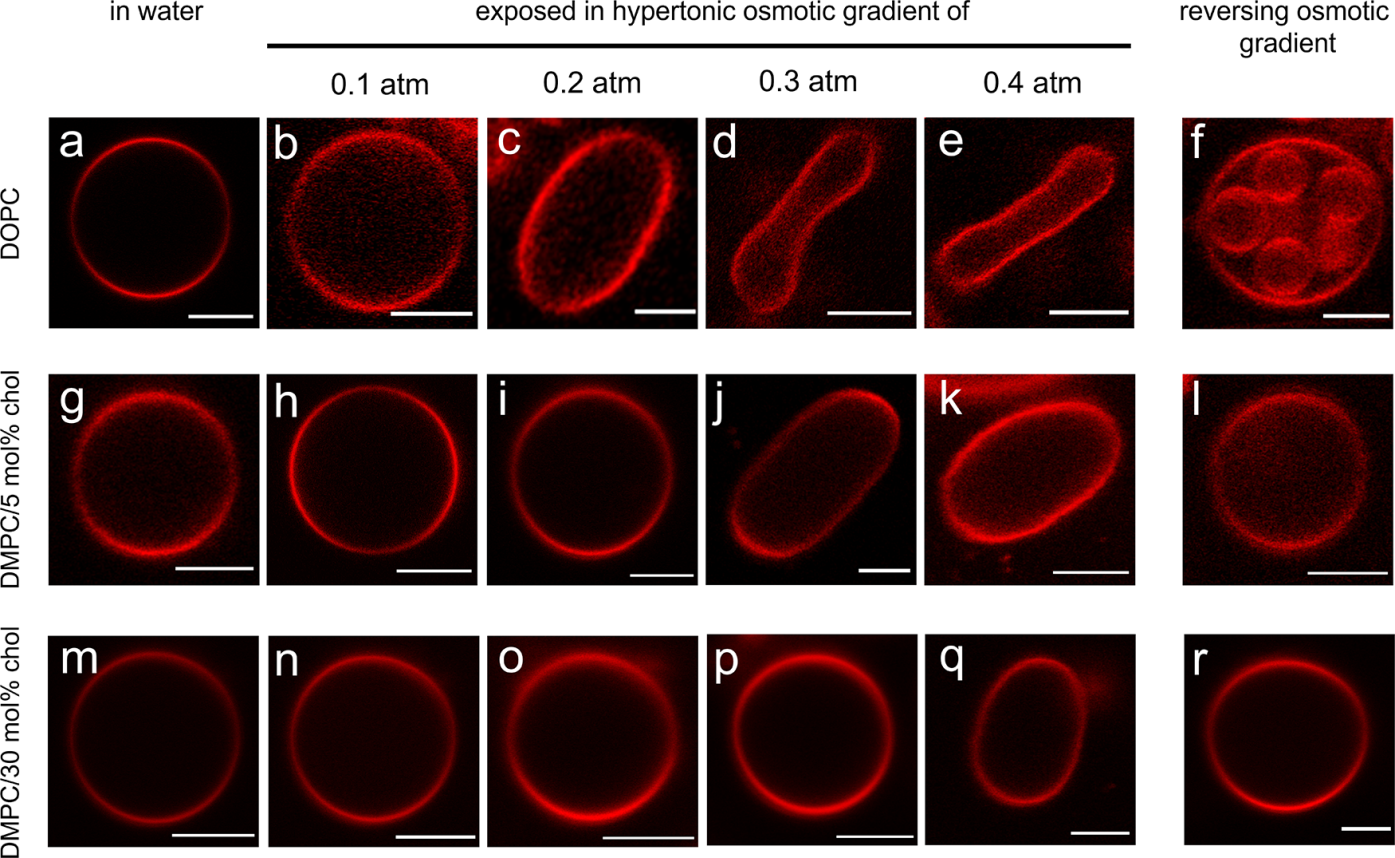
2D CLSM images of GUVs composed of DOPC (a–f), DMPC with 5 mol % cholesterol (g–l), DMPC with 30 mol % cholesterol (m–r), exposed to pure water (a,g,m) and osmotic gradients of π = 0.1 atm (b,h,n), 0.2 atm (c,i,o), 0.3 atm (d,j,p), and 0.4 atm (e,k,q). The GUVs exposed to an osmotic gradient of 0.4 atm were subsequently rinsed with pure water (f,l,r). The temperature was kept constant at 20 °C (DOPC) or 28 °C (DMPC/chol). The figure shows representative images for the different conditions and does not depict the same vesicle at different steps of the experiments. The scale bars represent 5 μm.

These observations provide the answer to Question 1 above. In a mechanical picture, the osmotic pressure of the outside solution acting to reduce the volume of the vesicle is balanced by a negative pressure inside the vesicle. Using the terminology of cell biology, the buckling of the membrane creates a negative turgor pressure. Without a major deformation, the GUVs can balance a pressure of 0.1 atm, which is experimentally relevant, albeit much smaller than the osmotic pressure of physiological saline of around 7 atm.

Figure 2 shows that, for small osmotic stresses, the vesicles retain their apparent spherical shape, while for higher values of π, there is a transition to a globally deformed state. To address Question 2 concerning the nature of this transition, we measured how the apparent aspect ratio of the vesicles depends on the osmotic stress. Since the experimental images are two-dimensional projections of three-dimensional objects, the measured aspect ratio is normally smaller than the true value. Nevertheless, as seen in Figure 3, there is a clear jump in the measured aspect ratio from a value of unity at bulk osmotic pressures up to 0.15 atm, to values >2 at π ≥ 0.2 atm. The answer to Question 2 is thus that there is a qualitative change in the way that the GUVs accommodate the volume reduction resulting from an increase in osmotic stress.

**Figure 3:**
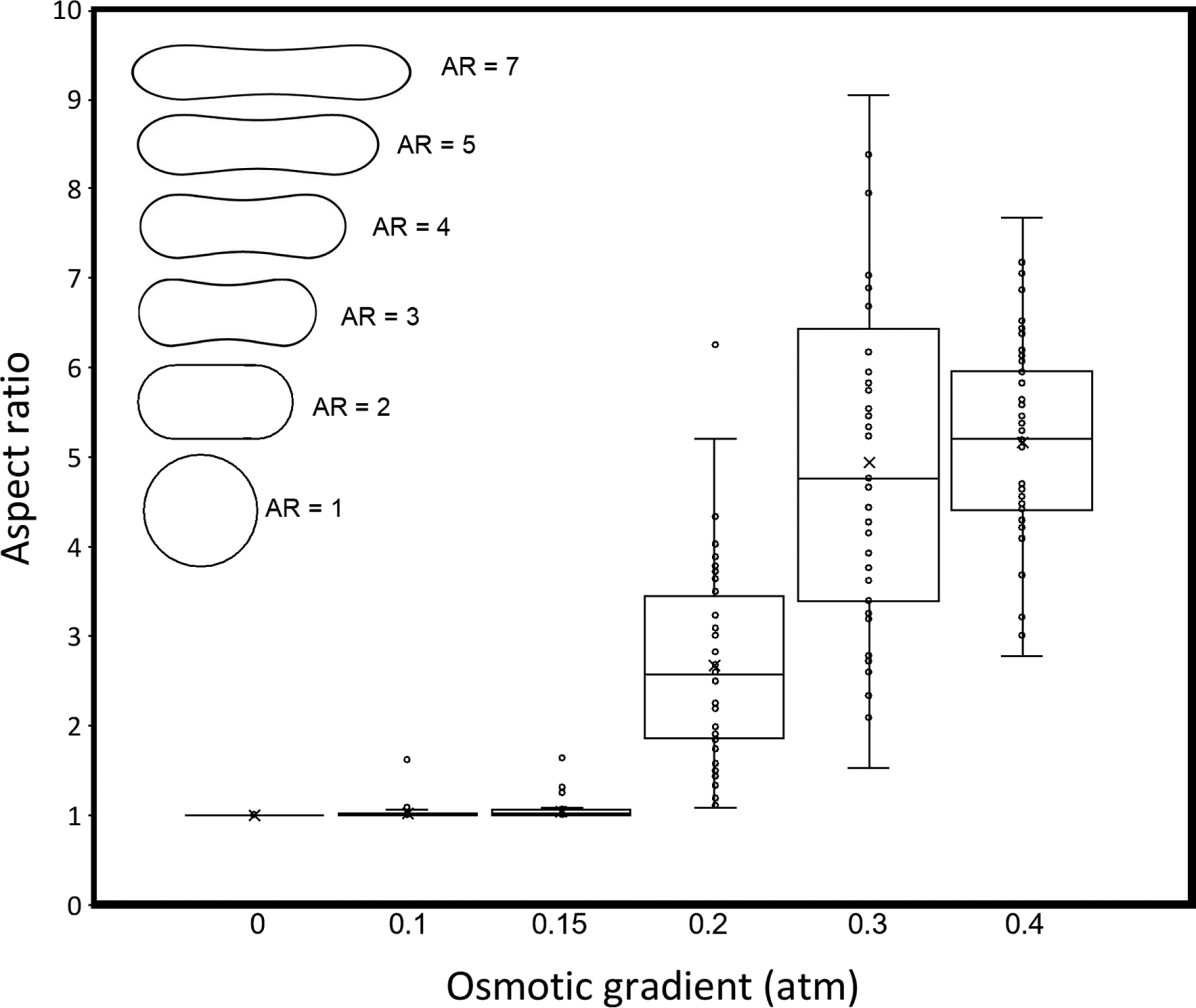
Box-whisker plots of the GUV aspect ratio (AR), defined as the ratio between the long axis (*L*_*L*_) and short axis (*L*_*S*_), measured for the 2D projected DOPC vesicles formed in water and then exposed to varying bulk osmotic pressures as indicated. The DOPC vesicle has a spherical shape (AR = 1) for π < 0.2 atm. For higher osmotic gradients, the DOPC vesicles adopt a prolate shape, becoming increasingly deformed with increasing magnitude of the osmotic gradient. The aspect ratio was measured for 50 individual vesicles for each condition (data points indicated by dots). Since the vesicle deformation increases abruptly with vesicle size for very large vesicles (Figure S7), we excluded the rare cases (7 out of approximately 100 vesicles) for which the corresponding undeformed vesicles have a diameter >15 μm. The inset shows schematic images of vesicles of various aspect ratios.

To interpret these results in more detail, we follow Seifert^50^ and expand the vesicle shape around a spherical shell of radius *R*_0_ in terms of the spherical harmonics *Y*_*lm*_
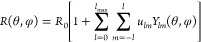
7Here, the expansion coefficients *u*_*lm*_ control the amplitudes of the modes *l,m*. The upper cutoff *l*_max_ in the expansion is determined by the fact that, at length scales comparable to the bilayer thickness, the description of the bilayer as a continuous, elastic surface is no longer relevant. Based on this description of shape fluctuations, Zhong-can and Helfrich^32^ analyzed the problem of vesicle deformation due to an external pressure. To leading order in *u*_*lm*_ and ignoring the effect of thermal fluctuations, they found that there is a linear instability in the axially symmetric *l* = 2, *m* = 0 mode at a critical pressure difference *Δp* given by^32^
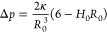
8Ignoring the spontaneous curvature term, the predicted instability occurs for Δ*p* ≈ 10^–2^ Pa when κ ≈ 20 *kT* and *R*_0_ ≈ 5·10^–6^ m. This corresponds to an osmolyte concentration of 5 nM, which is 6 orders of magnitude smaller than the concentration where we observe visible deformations. While glucose addition has been shown to induce an asymmetry in the bilayer, leading to a nonzero value of *H*_0_,^57^ this effect is likely negligible below concentrations in the millimolar range, since it is proportional to the osmolyte concentration.^58^ Thus, the spontaneous curvature terms in eq 8 are unlikely to be responsible for this large difference. Even though our experimental results are clearly not in quantitative accordance with the predicted threshold pressure, the qualitative phenomenology is well-reproduced in that it points to the existence of two regimes separated by a sharp transition. The theoretical analysis at *T* = 0 assumes that the membrane is laterally incompressible, which implies that, for small external pressures, there is a compensating lateral pressure in the membrane leaving the membrane unchanged. In contrast, a thermally excited bilayer can respond to a lateral pressure by a small increase in the (fluctuating) bending amplitude of all modes. At constant area, this results in a small decrease of the enclosed volume.^50^ As the mismatch between the bilayer area and optimal vesicle volume grows, a lateral pressure builds up, and eventually, a deformation analogous to the one predicted by Zhong-can and Helfrich sets in,^32,50^ and there is a large change in the amplitude of the *l* = 2, *m* = 0 mode resulting in a global deformation into a prolate shape as shown in Figure 1e,h.^45^

It is clear from Figure 3 that there is a considerable variation of the observed aspect ratios for a given external osmotic pressure above 0.2 atm. Part of this effect can be attributed to the problem of a projection of a 3D object to 2D. It is also clear that some of the variation is caused by a difference in size between the vesicles, where the larger vesicles are on average more deformed than the smaller ones (Figure S7). According to eq 8, there is a strong size dependence in how vesicles respond to an external pressure, and the observations on which Figure 3 is based are thus in qualitative agreement with theoretical predictions.

*Reversing the Osmotic Gradient*. After the GUVs were equilibrated with the bulk glucose solution, the bulk environment was exchanged back to pure water. As shown in Figure 1f, for pure DOPC vesicles exposed to the highest osmotic stress (π = 0.3 atm), daughter vesicles filled with practically pure water formed inside the GUVs. The same phenomenon was observed in DOPC vesicles with no added lipid dye, confirming that the results are not influenced by the small amount of fluorescent lipid analogue present in the membrane (Figure S4). It follows from the discussion in the previous section that water molecules inside GUVs previously exposed to a glucose solution are at a lower water chemical potential than those in the pure bulk medium due to the contribution from the excess membrane bending. When rinsing these vesicles in water, the external medium thus triggers a diffusion of water molecules across the bilayer into the GUVs. Even though this is a relatively fast process, the formation of daughter vesicles appears even more rapid. Furthermore, as demonstrated in Figure 1i, the addition of melittin, which makes the bilayer more permeable to water, eliminates the formation of daughter vesicles. The combined data in Figure 1f,I thus demonstrates the existence of a competition between molecular water diffusion and vesicle fission.

In the analysis of daughter vesicle formation, we identify two separate aspects: the *thermodynamic* driving force and the *kinetics* of the fission process. The analysis of both benefits from the fact that we are dealing with a relatively simple two-component lipid–water system. We begin by considering the thermodynamics of the fission process. In the deformed state, the chemical potential of water inside the vesicle is given by eq 5, and the vesicle volume is considerably smaller than the value 4*πR*_0_^3^/3 of the initially formed GUV. When a daughter vesicle of radius *R*_*D*_ is formed either inside or outside the GUV, the volume enclosed by the GUV is changed by an amount Δ*V*_0_ = ±4*πR*_*D*_^3^/3, where the positive sign applies to endocytosis and the negative sign applies to exocytosis. The area of the mother GUV has in both cases changed by an amount Δ*A* = −4*πR*_*D*_^2^. The change in curvature free energy in the process comes both from the formation of a new, unconstrained vesicle and from changes in the area and volume of the GUV. Assuming small Δ*V*_0_ and Δ*A*, the change in bending free energy can be expressed as
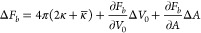
9Thus, the formation of a new vesicle generically involves an increase in bending free energy of 4π(2κ + κ̅), which for typical values of the bending constants (κ ≈ 20 *kT*, κ̅ ≈ −5 *kT*)^59^ amounts to more than 300 *kT*. This free energy penalty can be reduced if there is a bilayer asymmetry so that there is a preferred curvature^60^ that matches the vesicle radius, caused by protein binding or an uneven distribution of lipids between the two leaflets.^11,14^ The term containing Δ*A* is negative for an initially buckled bilayer, since an area decrease involves a release of the stress on the bilayer. As discussed above, Δ*V*_0_ can be positive or negative depending on whether the new vesicle is formed inside or outside the GUV, while the volume derivative is given in eq 6 and is negative. Thus, the free energy difference between forming a daughter vesicle through endocytosis or exocytosis is
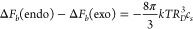
10For a daughter vesicle of radius 1.5 μm and a glucose concentration of 10 mM, this free energy difference is of order −10^8^*kT*, demonstrating that the formation of daughter vesicles *inside* the mother GUV is strongly preferred relative to the outside. The large value of the free energy reflects the fact that the free energy gain in incorporating more than 10^11^ water molecules in one process is much larger than doing it one by one through diffusion, a process which involves a free energy change of order −10^–3^*kT* per water molecule.

Based on the estimates made for the free energy in eq 10, it follows that the right-hand side of eq 9 is strongly negative (<−10^7^*kT*) for realistic values of the radius *R*_*D*_ of the daughter vesicle. However, for vesicles exposed to osmotic stresses below the global deformation limit, Δ*V*_0_ becomes small enough that it only allows for the formation of very small daughter vesicles, so that the first term on the right-hand side of eq 9 becomes significant. The most direct explanation of why daughter vesicles do not appear for these systems is thus that the process is not thermodynamically favorable, which provides an answer to Question 5 above. We conclude that the formation of internal daughter vesicles is thermodynamically strongly favored for vesicles that are visibly deformed. This process is significantly more favored than the process leading to external daughter vesicles, which answers Question 4. However, for the formation of daughter vesicles to occur, the process must also be *kinetically* allowed, and in the next paragraph, we address this aspect of the fission process.

It is a well-established fact that pure DOPC lipid vesicles, as well as lipid vesicles in general, remain stable relative to both fusion and fission in homogeneous aqueous media. However, in the experiments illustrated in Figure 1, the osmotic pressure is higher inside the vesicles than in the bulk, and as discussed above, this results in a thermodynamic bias for the fission process for vesicles large enough to overcome the first term in eq 9 while being smaller than the volume of the mother vesicle. This leaves a large window of thermodynamically allowed sizes of the resulting daughter vesicles, while in experiments, the observed daughter vesicles have a narrow size distribution, as shown in Figure 4. Thus, we conclude that the size of the daughter vesicles is instead determined by the kinetics of the fission process. Following Kozlov and Markin,^61^ one can identify three stages of the fission process: (*i*) an invagination of the vesicle bilayer leading to the formation of a neck, (*ii*) a hemifusion between the outer leaflets of the neck, resulting in the water being enclosed in a new compartment separated from the bulk solution, and (*iii*) a second hemifusion leading to a separate daughter vesicle on the inside of the GUV. In the absence of an osmotic gradient (i.e., for a spherical vesicle), the first step involves a substantial increase in curvature energy. For a partially collapsed mother vesicle, e.g., due to a large osmotic imbalance, this free energy barrier is greatly reduced, since the bilayer is already bent and the localization of this buckling to an invagination is relatively cheap from an energetic perspective. As shown in Figure 4b–g, the daughter vesicle size is, within the experimental accuracy, independent of the initial osmotic imbalance as long as it is large enough to cause a global deformation of the mother vesicle. This observation indicates that the rate-limiting step of the fission occurs at the initial invagination step. The fact that the daughter vesicles are relatively large is furthermore consistent with the prediction that long-wavelength deformations, corresponding to small values of *l* in eq 7, are associated with lower energy costs compared to short-wavelength ones.

**Figure 4:**
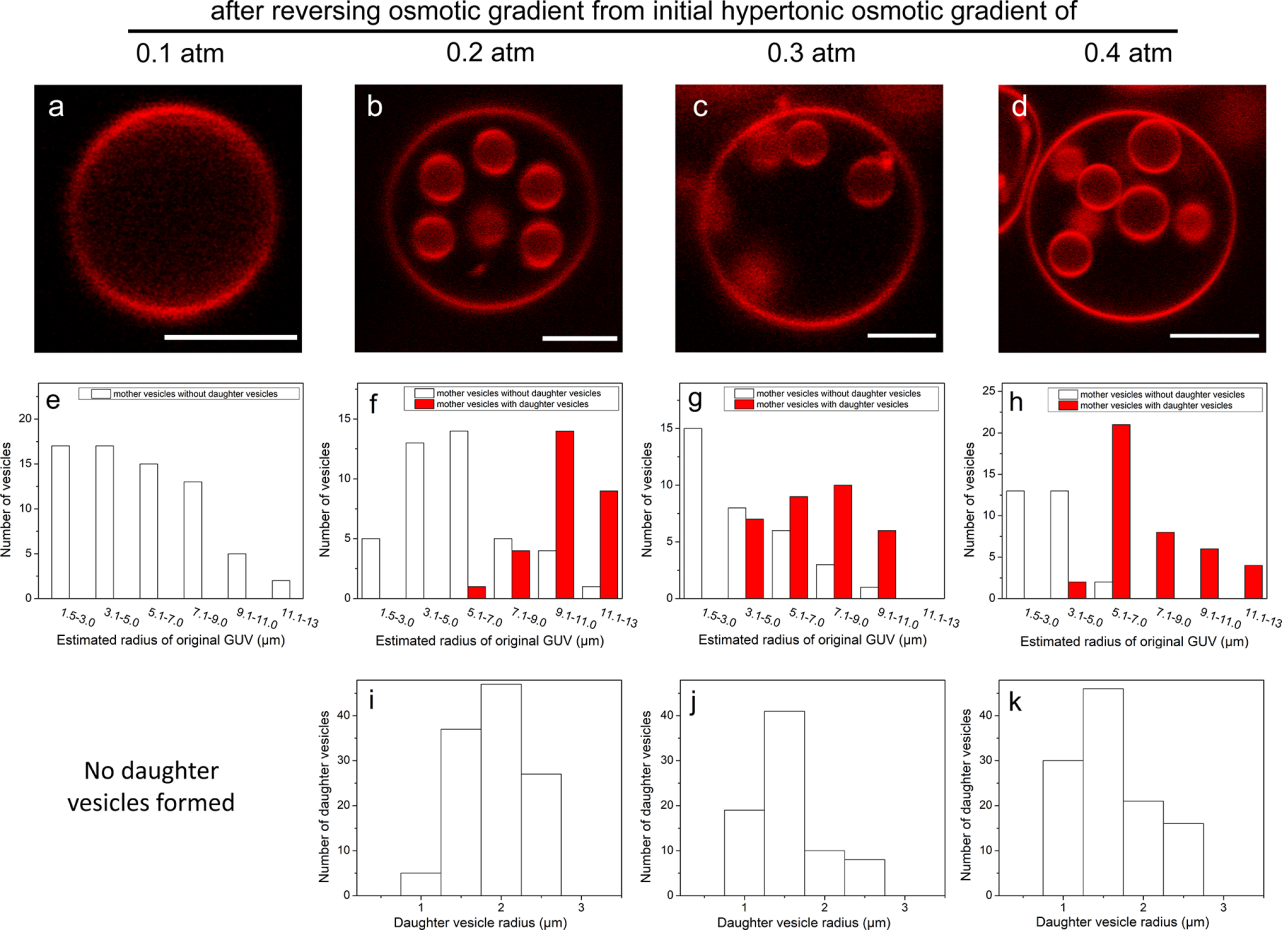
2D CLSM images of GUVs composed of DOPC (20 °C) after reversing the osmotic gradient from initial values of π = 0.1 atm (a), π = 0.2 atm (b), π = 0.3 atm (c), and π = 0.4 atm (d). The scale bars represent 5 μm. When the gradient is reversed from 0.1 atm, no daughter vesicles are observed. For the higher osmotic gradients, daughter vesicles are formed inside the larger vesicles. Panels (e–g) show the occurrence of daughter vesicles for mother vesicles of different original size, here represented by the radius of the original spherical vesicles, as recalculated from the estimated total membrane area including both mother and daughter vesicles. Panels (i–k) show histograms of the daughter vesicle radius under conditions corresponding to panels (b–d). For each condition, 35 mother vesicles containing daughter vesicles were analyzed.

In conclusion, we have demonstrated how GUV vesicles can obtain osmotic equilibrium with an outside medium of initially higher osmotic pressure by bending the lipid bilayer, creating the analogue of a negative turgor pressure. For small differences in the osmotic pressure, the resulting deformations are small enough to not be detected by visible light, while for larger differences in osmotic pressure, there is an abrupt transition to a regime where the vesicles undergo a global deformation. The existence and nature of this deformation is qualitatively in accordance with previous theoretical and computational predictions,^32,33^ although the values of the osmotic pressure where the transition occurs (0.1 to 0.2 atm) are many orders of magnitude larger than predicted by the bare bending energy of a spherical shell. We furthermore found that the critical osmotic pressure is approximately proportional to the bending rigidity of the bilayer, supporting our interpretation that the deformation should be understood as a balance between bending energy and osmotic stress.

When reversing the osmotic gradient for a globally deformed vesicle, we found reproducible formation of internal daughter vesicles of well-defined size. In the ensuing thermodynamic analysis, we showed that this internal fission process is connected to a sizable gain in free energy. For a pure lipid vesicle, where water diffusion across the membrane is relatively slow, this fission process is clearly rapid enough to compete with water diffusion across the bilayer.

Our results provide novel insights into the deformation and fission of vesicles in response to small and moderate osmotic gradients. It is shown that the global deformation of the spherical vesicle occurs at osmotic pressure differences of approximately 0.1 atm. This finding is in stark quantitative contrast to theoretical predictions for the stability of spherical vesicles under osmotic stress (eq 8), which have predicted the bending energy to give a negligible contribution for all practical purposes. The fundamental origin of the apparent difference is yet unclear to us. However, since all our experiments were performed in bulk, we can rule out artifacts due to the interaction with solid surfaces and boundaries. Furthermore, any small presence of contaminant is unlikely to be responsible for the discrepancy, as its bulk concentration would need to be in the millimolar concentration range to balance the outside osmotic pressure. A theoretical understanding of the osmotic stability of vesicles beyond the harmonic treatment underlying eq 8 is one possible route toward a deeper understanding of our experimental results. If correct, these have large potential impact also on the understanding of osmotic regulation in living cells, as well as fission and fusion processes. Our findings imply that, even without a developed cell wall, the bending energy of the lipid membrane itself is sufficient to withstand significant mismatches in osmotic pressure. The primary response to such a mismatch is a small, local buckling of the cell membrane leading to a decrease in volume that is significantly less drastic than the one needed to equalize the osmolyte concentration. At larger osmotic gradients, the cell deforms on a larger scale to partially balance the external osmotic pressure by a negative turgor pressure.

The rapid, reproducible formation of daughter vesicles inside the GUV upon reversal of the osmotic gradient shows that the endocytosis process is strongly thermodynamically favored for these conditions. Furthermore, our thermodynamic analysis showed that it requires significant bending free energies stored in the bilayer deformations to be kinetically allowed. Most previous studies of vesicle fusion and fission phenomena, whether in pure lipid systems^15,16,21^ or assisted by specific proteins in living cells,^3−7,62^ concern the kinetic aspects of the fission process, while the present study additionally sheds light on the somewhat less studied thermodynamic aspects of these processes. In particular, we have demonstrated that, from a geometrical perspective, the fission of daughter vesicles from a mother vesicle with a symmetrical bilayer is only thermodynamically allowed when the mother vesicle is significantly deformed from spherical shape. Thus, if there is a sizable temporary osmotic imbalance, there can be a very large energetic driving force for a fission process. Second, even at equilibrium with respect to water transport, large free energies can be stored in excited local bending modes, which can be used to drive fission or fusion processes. Relating these basic findings to the formation of daughter vesicles in living systems^14,63−67^ remains an important topic for future investigations.

## Supporting Information Available

Materials and methods; overview figures for DOPC vesicles at different osmotic gradients (Figure S1), free daughter vesicles formed after reversing osmotic gradient (Figure S2), DOPC GUV membrane with pores induced by melittin (Figure S3), daughter vesicles formed in unlabeled DOPC GUVs (Figure S4), deformation by osmotic gradients generated by adding PEG2000 to the outside solution (Figure S5), leakage test of deformed vesicles (Figure S6), size-dependent osmotic response of DOPC vesicles (Figure S7) (PDF)
Movie of the dynamics of daughter vesicles, showing that these are fully separated from the mother vesicle (AVI)
Movie of the dynamics of daughter vesicles, showing that these are fully separated from the mother vesicle (AVI)

## Supplementary Material

jz1c03369_si_001.pdf

jz1c03369_si_002.avi

jz1c03369_si_003.avi
