# Supplementary material for: Vesicles Balance Osmotic Stress with Bending Energy That Can Be Released to Form Daughter Vesicles
Source: J Phys Chem Lett. 2022 Jan 10;13(2):498–507. doi: 10.1021/acs.jpclett.1c03369 (PMC8785185; doi:10.1021/acs.jpclett.1c03369)
Supplement: Supplementary file 1 — jz1c03369_si_001.pdf [file jz1c03369_si_001.pdf]

Supporting Information
for
Vesicles Balance Osmotic Stress with Bending Energy That Can Be Released to Form Daughter Vesicles

Xiaoyan Liu, Joakim Stenhammar, Håkan Wennerström and Emma Sparr*

Physical Chemistry, Lund University, 221 00 Lund, Sweden

1. Materials and methods

Materials. 1,2-dioleoyl-sn-glycero-3-phosphocholine (DOPC), 1,2-ditetradecanoyl-sn-glycero-3-phosphocholine (DMPC), 1,2-dioleoyl-sn-glycero-3-phosphoethanolamine-N-(lissamine rhodamine B sulfonyl) (ammonium salt) (Liss Rhod PE) were purchased from Avanti Polar Lipids. Glucose (purity, ⩾99.5%), Polyethylene glycol 2000 (PEG2000), cholesterol (purity, >99%), chloroform (analytical grade, ⩾ 99.8%), methanol (analytical grade, ⩾99.9%) were purchased from Sigma-Aldrich. Alexa Fluor 488 N-hydroxysuccinimidyl ester, (Alexa Fluor™ 488 NHS Ester, $C_{25}H_{15}Li_2N_3O_{13}S_2$) was purchased from Thermo Fisher Scientific Inc. Milli-Q water was used in all the experiments.

GUV preparation. GUVs were prepared on indium tin oxide (ITO)-coated coverslips (30-60 Ohms/Sq, Sigma-Aldrich) by the electroformation method[1, 2] in a fluidic flow channel.[2] By using this fluidic flow channel we can easily exchange solutions and also observe free vesicles that do not attach or sediment on surfaces, which excludes phenomena induced by surface adhesion. Stock solutions of either DOPC or DMPC with 5 mol% cholesterol, or DMPC with 30 mol% cholesterol were prepared in chloroform/methanol (9:1 volume ratio) at a concentration of 0.2 mg/ml. To all lipid samples, 0.5 mol% the fluorescent lipid analogue Liss Rhod PE (red) was added. To prepare GUVs, the ITO-coated coverslips were first cleaned with ethanol and dried by nitrogen gas. 10 μL lipid solution was then deposited onto the conductive side of the ITO-coated coverslip and dried in a vacuum chamber overnight. The lipid coated coverslip was then mounted to the adhesive underside of a microchannel (Ibidi sticky-Slide VI 0.4). Another ITO-coated coverslip was attached to the top side of the microchannel with the conductive side towards sample solution. Next, conductive wires were used to connect the conductive sides of the two ITO-coated coverslips to the electrodes from the frequency generator. The AC electric field (10 Hz, 3V) was applied for 3 hours to generate the GUVs. The GUVs were prepared in the fluid state, DOPC at 20 °C, DMPC with cholesterol at 28 °C. DOPC GUVs were prepared in the liquid disordered phase, and DMPC/chol GUVs in the liquid ordered phase.[3]

Experimental procedure. GUVs composed of either DOPC or DMPC/chol were prepared using the electroformation method. After completing the vesicle preparation, the GUVs were observed by CLSM. The preparation of GUVs and CLSM observation were carried out at 20 °C (DOPC) or 28 °C (DMPC/chol). Hypertonic osmotic gradients were generated by adding glucose or PEG2000 stock solutions to the fluidic flow channel with the prepared GUVs. Typically, 6.5-29 μL of 100 mM glucose were added to the sample (150 μL) to generate an osmotic gradient of 4-16 mM (0.1-0.4 atm). For proper mixing after injection, the fluidic flow channel cell was gently rotated several times. Due to the mixing, it was typically not possible to image the same vesicle at all steps of the experiments, and all figures therefore show representative images for the different conditions. After this, the two wells of the fluidic channel were sealed by parafilm to prevent water evaporation. The sample was then observed by using CLSM. For each condition investigated (step-wise increase in osmotic gradient and then addition of water at a fixed osmotic gradient) the experiment was repeated at least 3 times.

2. Confocal Laser Scanning Microscope.

The fluorescent GUVs were observed by confocal laser scanning microscope (CLSM, Leica SP5) operated in the inverted mode (D6000I). The temperature of the samples was controlled with an accuracy of 0.2 °C by mounting the CLSM to a thermostated enclosure. Samples were equilibrated for 2 hours at 20 or 28 °C before observation. The red fluorescence of the membrane lipid analogue (Liss Rhod PE) and the green fluorescence probe, Alexa Fluor™ 488 NHS Ester (Alexa488), were excited by using a HeNe laser at 543 nm and an argon-ion laser at 488 nm, respectively.

A time-lapse sequence of confocal fluorescence images (Movie S1) and the image stack from top-to-bottom *z*-axis scans (Movie S2) clearly shows that the daughter vesicles formed inside DOPC GUVs are separated from the mother vesicle.

3. Movies

Movie S1. A time-lapse sequence of confocal fluorescence images of Liss Rhod PE labeled DOPC vesicle (red) after reversing osmotic gradient by rinsing with water and the fluorophore Alexa488 (green).

Movie S2. Top to bottom *z*-axis scans of an unlabeled DOPC vesicle after reversing the osmotic gradient by rinsing with water and the fluorophore Alexa488 (green).

4. Supplementary figures

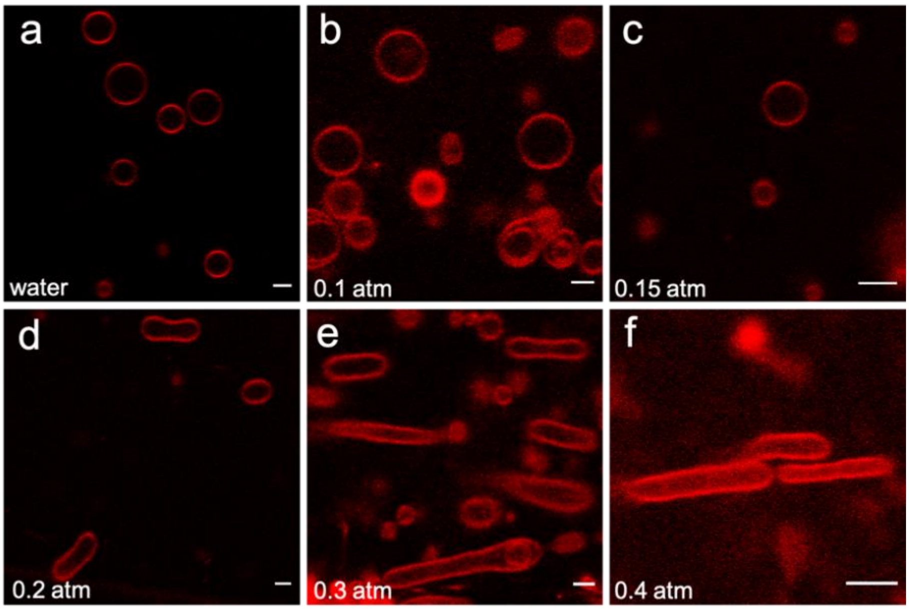

Figure S1. Vesicle deformation at different osmotic gradients. 2D CLSM images of DOPC vesicle in pure water (a), and exposed to osmotic gradients of 0.1 atm (b), 0.15 atm (c), 0.2 atm (d), 0.3 atm (e), and 0.4 atm (f), all at $T = 20$ °C. The scale bar represents 5 μm.

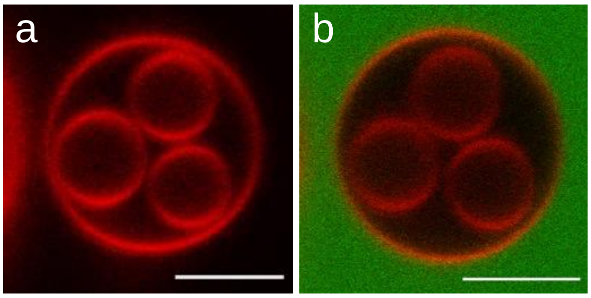

Figure S2. Daughter vesicles are fully disconnected from the outside solution. The water-soluble green dye was added after the daughter vesicles were already formed, showing no detectable green fluorescence inside the daughters. The 2D CLSM images of DOPC GUVs (red) were obtained (a) after reversing the osmotic gradient from an initial value of 0.3 atm, and (b) after subsequent addition of Alexa488 (green) to the outside solution. The scale bar represents 5 µm.

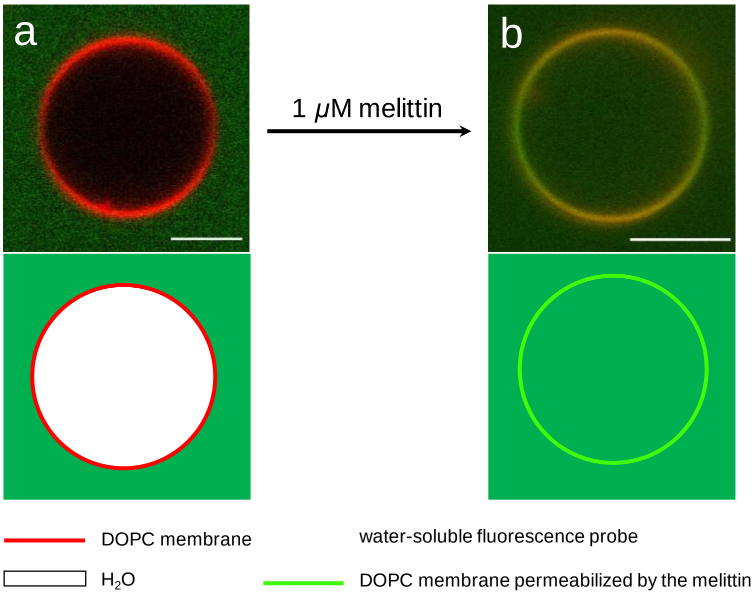

Figure S3. Melittin causes membrane leakage. 2D CLSM images of (a) a DOPC vesicle (Liss Rhod PE labeled, red) with the outer fluid labelled by the green fluorophore Alexa488, (b) a DOPC vesicle subjected to 1 µM melittin, both at T = 20 °C. The scale bar represents 5 µm.

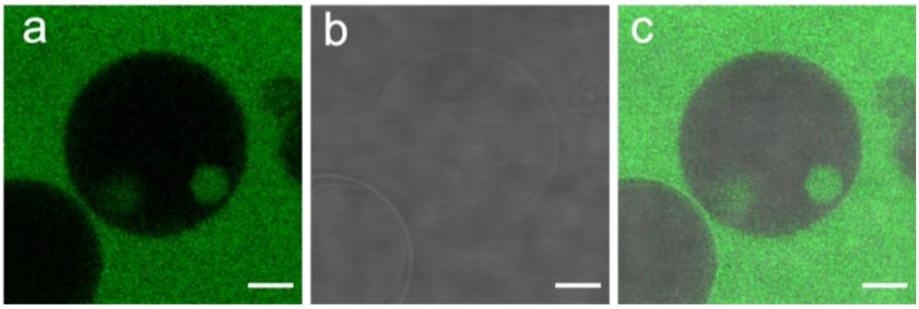

Figure S4. The formation of daughter vesicles is not influenced by the presence of small amounts of a fluorescent lipid analogue. The panels show (a) fluorescence image, (b) bright field image, and (c) merged image of the same unlabeled DOPC GUV after reversing the osmotic gradient from an initial value of 0.3 atm. The green fluorophore Alexa488 was added to the rinsing water to label the outside liquid. T = 20 °C. The scale bar represents 5 µm.

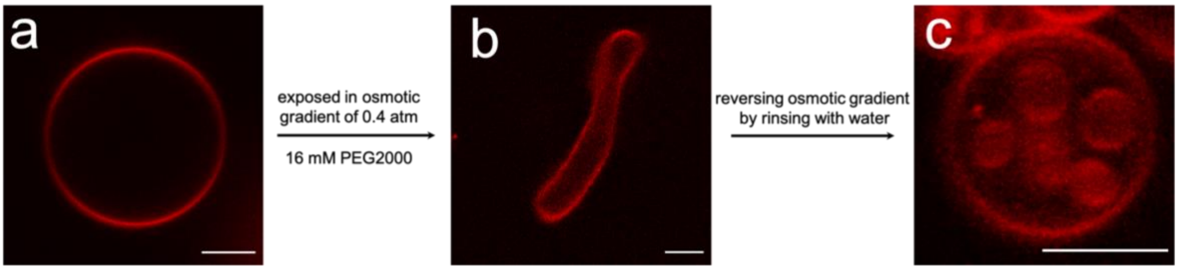

Figure S5. Response to osmotic gradient generated by adding PEG2000 to the outside solution instead of glucose. 2D CLSM images of DOPC GUVs (a) exposed to pure water, and (b) in an osmotic gradient of 0.4 atm (16 mM PEG2000). (c) shows a DOPC vesicle that has first been exposed to an osmotic gradient of 0.4 atm and subsequently rinsed by pure water to reverse the gradient. T = 20 °C. The scale bar represents 5 μm.

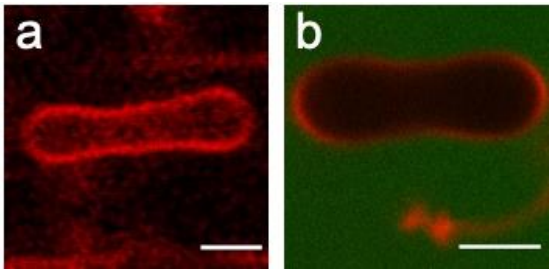

Figure S6. Absence of leakage across the lipid membrane of deformed vesicles. 2D CLSM images of DOPC GUVs (red) (a) exposed to an osmotic gradient of 0.3 atm, (b) after addition of Alexa488 (green) to the outside solution. The absence of green dye inside the deformed vesicle in (b) shows the absence of significant water leakage through the deformed vesicle. T = 20 °C. The scale bar represents 5 μm.

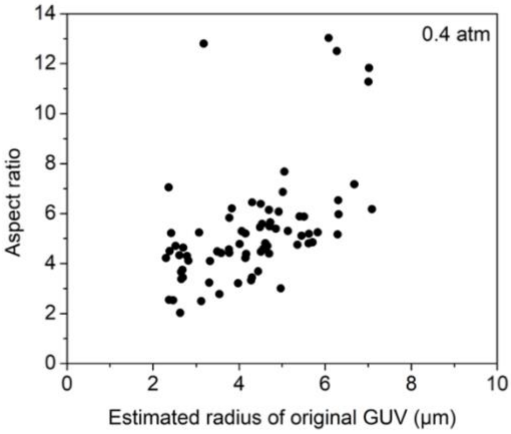

Figure S7. Aspect ratio between the long (L_L) and short axis (L_S) of prolate DOPC vesicles present at 0.4 atm for vesicles of different sizes. The membrane area of the prolate vesicles were calculated using Eq.S1. Assuming that the membrane area is unchanged upon deformation, the corresponding radius of the original spherical vesicles was then calculated. 70 vesicles were analyzed.

$$A = \frac{\pi L_S^2}{2}\left(1 + \frac{L_L}{L_S e}\arcsin e\right); \quad e \equiv \sqrt{1 - \frac{L_S^2}{L_L^2}} \qquad \text{(S1)}$$

References

(1) Angelova, M. I.; Dimitrov, D. S. Liposome electroformation. *Faraday Discuss. Chem. Soc.* **1986,** *81*, 303-311.
(2) Wang, M.; Mihut, A. M.; Rieloff, E.; Dabkowska, A. P.; Månsson, L. K.; Immink, J. N.; Sparr, E.; Crassous, J. J. Assembling responsive microgels at responsive lipid membranes. *Proc. Natl. Acad. Sci. U. S. A.* **2019,** *116,* 5442-5450.
(3) Almeida, P. F. F.; Vaz, W. L. C.; Thompson, T. E. Lateral diffusion in the liquid phases of dimyristoylphosphatidylcholine/cholesterol lipid bilayers: a free volume analysis. *Biochemistry* **1992,** *31*, 6739-6747.
